# Glutamatergic Neurotransmission from Melanopsin Retinal Ganglion Cells Is Required for Neonatal Photoaversion but Not Adult Pupillary Light Reflex

**DOI:** 10.1371/journal.pone.0083974

**Published:** 2013-12-31

**Authors:** Anton Delwig, Sriparna Majumdar, Kelly Ahern, Matthew M. LaVail, Robert Edwards, Thomas S. Hnasko, David R. Copenhagen

**Affiliations:** 1 Department of Ophthalmology, University of California San Francisco, San Francisco, California, United States of America; 2 Department of Anatomy, University of California San Francisco, San Francisco, California United States of America; 3 Department of Physiology, University of California San Francisco, San Francisco, California, United States of America; 4 Department of Neurology, University of California, San Francisco San Francisco, California, United States of America; 5 Department of Neurosciences, University of California San Diego, San Diego, California, United States of America; Morehouse School of Medicine, United States of America

## Abstract

Melanopsin-expressing retinal ganglion cells (mRGCs) in the eye play an important role in many light-activated non-image-forming functions including neonatal photoaversion and the adult pupillary light reflex (PLR). MRGCs rely on glutamate and possibly PACAP (pituitary adenylate cyclase-activating polypeptide) to relay visual signals to the brain. However, the role of these neurotransmitters for individual non-image-forming responses remains poorly understood. To clarify the role of glutamatergic signaling from mRGCs in neonatal aversion to light and in adult PLR, we conditionally deleted vesicular glutamate transporter (VGLUT2) selectively from mRGCs in mice. We found that deletion of VGLUT2 in mRGCs abolished negative phototaxis and light-induced distress vocalizations in neonatal mice, underscoring a necessary role for glutamatergic signaling. In adult mice, loss of VGLUT2 in mRGCs resulted in a slow and an incomplete PLR. We conclude that glutamatergic neurotransmission from mRGCs is required for neonatal photoaversion but is complemented by another non-glutamatergic signaling mechanism for the pupillary light reflex in adult mice. We speculate that this complementary signaling might be due to PACAP neurotransmission from mRGCs.

## Introduction

Melanopsin-expressing retinal ganglion cells (mRGCs) in the eye mediate many light-evoked non-image forming functions including neonatal photoaversion [1], [2] and the adult pupillary light reflex (PLR) [3], [4]. Both glutamatergic and peptidergic neurotransmission mechanisms have been postulated to relay visual signals from mRGCs to their neuronal targets in the brain [5], [6]. However, the role of these neurotransmitters for individual non-image forming responses remains poorly understood.

Glutamatergic synaptic transmission requires the sequestration of glutamate into presynaptic vesicles. One of three isoforms of the vesicular glutamate transporter, VGLUT1, VGLUT2 or VGLUT3, is essential for filling vesicles in glutamatergic neurons (reviewed in [7]). Individual classes of neuron almost always express a single VGLUT isoform. Retinal ganglion cells (RGCs), the projecting output neurons of the retina, stain exclusively with VGLUT2 antibodies and express VGLUT2 mRNA [8]–[10]. Prior studies of glutamatergic neurotransmission from retinal ganglion cells to the thalamus and between midbrain neurons in the ventral tegmental area demonstrated that conditional deletion of VGLUT2 abolishes evoked synaptic release of glutamate from these neurons [11], [12]. Thus, loss of VGLUT2 expression in mRGCs would be expected to abolish light-activated glutamatergic signaling from mRGCs.

MRGCs also express pituitary adenylate cyclase-activating polypeptide (PACAP), which is present in the retina before birth [5], [13] and co-localizes with VGLUT2 in mRGC projections in the brain [6]. PACAP signaling occurs on a slower timescale [14] but, in principle, could mediate many light-elicited non-image forming functions that often occur over extended periods of time (seconds to hours).

In neonatal mice, light evokes aversive responses including negative phototaxis and distress ultrasonic vocalizations [1], [2]. Until postnatal day 10 (P10), these responses are mediated by mRGCs, the only functional photoreceptors in the eye at this age [1], [2]. The extent to which retinofugal signal transmission from mRGCs relies on glutamatergic signaling in young neonates is not known.

The pupillary light reflex (PLR) in adult mice is mediated exclusively by signaling from mRGCs. Visual signals for the PLR can originate from intrinsic light activation of mRGCs themselves, or from light-activated rod and cone signals that synaptically drive the mRGCs. The necessity of mRGC-mediated neurotransmission is exemplified by the finding that selective destruction of mRGCs completely abolishes the PLR in mice [4]. However, the identities of the neurotransmitters used by mRGCs for the PLR in adults remain elusive. PACAP-null mice have a normal PLR [15] suggesting that glutamatergic signaling from mRGCs is sufficient. However, other studies suggest that PACAP contributes to the size and stability of the PLR. Mice that lack the main receptor for PACAP have smaller and less sustained pupillary responses to light [16].

To clarify the role of glutamatergic signaling from mRGCs for neonatal aversion to light and the adult PLR, we studied these non-image forming responses using conditional deletion of VGLUT2 in mRGCs in mice.

## Methods

### Ethics Statement

The University of California, San Francisco Institutional Animal Care and Use Committee (Animal Welfare Assurance Number: A3400-01) specifically approved this study. The protocols, animal care procedures and the experimental methods meet all of the guidelines on the care and use of laboratory animals by the U.S. Public Health Service.

### Animals

Mice were housed in an AALAC-accredited pathogen-free UCSF animal facility with *ad libitum* access to food and water, and with a 12-hour light-dark cycle with lights on at 7 AM and off at 7 PM.

Animals that were used in this study were bred following the approach of Hnasko *et al.* [12]. In the first step, mice homozygous for *opn4^cre^* (*opn4^cre^*/*opn4^cre^* [17]) were crossed with mice homozygous for floxed-*slc17a6* (*slc17a6^loxP^/slc17a6^lox^* [12]), which encodes VGLUT2, the vesicular glutamate transporter responsible for sequestering glutamate in the synaptic vesicles of the mRGC axons. In the second step, male progeny from this cross, who were all heterozygous for *opn4^cre^* and floxed- *slc17a6* (*opn4^cre^*/+; *slc17a6^loxP^*/+;), were backcrossed to females homozygous for floxed- *slc17a6.* On average, 25% of mice obtained from this second cross had one copy of *opn4^cre^* gene and one copy of the floxed *slc17a6* gene (*opn4^cre^*/+; *slc17a6^loxP^*/+; controls). 25% had one copy of *opn4^cre^* gene and two copies of the floxed *slc17a6* gene (*opn4^cre^*/+; *slc17a6^loxP^*/*slc17a6^loxP^*; conditional knockout, cKO). The other 50% of mice had no *opn4^cre^* expression (+/+;*slc17a6^loxP^*/+ or +/+; *slc17a6^loxP^*/*slc17a6^loxP^*) and were not used in the analysis. It should be noted that *opn4^cre^* allele is a knockin that replaces endogenous *opn4* gene. Therefore, control and cKO mice used in this study had only one copy of the melanopsin gene.

Ages of mice are described with the respective experiments. Neonates were postnatal day (P) 7–9; adults were P37–P289. Previous studies demonstrated that *opn4-cre*-mediated recombination emerges in the retina by embryonic day 15 [18]. This onset age is well before the ages when we tested for photoaversion and the PLR.

### Neonatal Behavioral Testing

All testing was performed during the subjective daytime. Animal behaviors were monitored with an infrared camera and an ultrasound detector. The infrared images were recorded at 10 frames per second onto a laptop computer using BTV Pro (Ben Software, http://www.bensoftware.com). Ultrasonic vocalizations (USVs) from mouse pups were recorded with an ultrasound detector (UltraSound Advice, UK; model: mini-3) and a sound recorder (Sony PCM-M10). Timing of USVs was detected offline by thresholding the root mean square levels (5 msec bins; Matlab) as described previously [2]. Movement of pups was quantified by frame differencing [19] as the number of pixels that changed their intensity value more than the threshold.

where *V(x,y,t)* is the 8-bit value of a pixel's intensity at location *x, y* and at time *t*. Threshold was determined for each individual recording as the difference between the darkest region of the background and the lightest region of the mouse’s body. The Matlab code used for analyses is available from authors upon request.

The monitoring chamber (10×3×4 cm; L×W×H) was made of clear acrylic warmed by a heating pad to 35°C. Using the same chamber, we previously showed that light evoked both negative phototaxis [1] and ultrasound vocalizations [2]. In the present experiment, we positioned LED light sources (Philips Lumileds Lighting Company; model: Luxeon III star, LXHL-LB3C, wavelength = 470 nm) at 5 cm from each end of the testing chamber. The measured power flux at each end of the chamber was 40 mW/cm^2^ (UDT Instruments, San Diego, CA; model S471). The calculated photon flux at 470 nm is 9×10^16^ photons/sec/cm^2^, which is roughly equivalent to the amount of blue light in the direct sunlight at midday. Taking into account that eyelids are closed at this age (about 100-fold attenuation of light [3]) and that pups are free to move inside the testing chamber (4-fold difference in light intensity depending on the location inside the chamber), we estimate that the amount of light that reached their corneas ranged from 100 to 400 µW/cm^2^ (2.2 to 9×10^14^ photons/sec/cm^2^).

Mice were kept in darkness for at least one hour before the experiment. Neonatal pups at ages P7 to P9 were tested individually, and transferred to the testing chamber under dim red light. Pups were allowed to acclimate to the chamber until the isolation-induced 62-kHz USVs calls ceased (10–15 min). Subsequently, a recording trial began with a 1-min baseline in the dark, a 1-min exposure to light, followed by an additional 1 min of recording in the dark. Movement and vocal responses were quantified as the amount of net movement (in kilo Pixels) and the number of 62-kHz vocalizations during each 1-min interval.

### Pupillary Light Responses

Adult mice were dark adapted for at least 60 min. Under dim red illumination, the dark-adapted, unanesthetized mice were placed in a rodent restrainer device and positioned in front of an infrared video camera. A blue (480 nm) light stimulus was delivered to the right eye and pupillary constriction in the left eye was monitored under infrared light. The infrared images were recorded onto a laptop computer using BTV Pro (Ben Software, http://www.bensoftware.com) at 30 frames per second. The videos were analyzed in NIH Image. The pupil diameter was manually measured once per two seconds (synchronized to the onset of light). A trial consisted of 1 min in the dark, 1 min in the light followed by 1 min in the dark. The data was plotted in Matlab and GraphPad Prism. The age of mice ranged from P44 to P266. The age had no discernible effect on the size of the PLR.

To confirm the ability of cKO mice to constrict their pupils, 5 ul of Pilocarpine HCl (1%; Bausch & Lomb) was applied to each eye. After a few minutes, excess fluid was wiped with a paper tissue. Recordings of pupil diameter were made in the darkness 20 min after the application of Pilocarpine. It should be noted that Pilocarpine’s effect has a slow onset and is short in duration. After 40 min, pupils started to re-dilate.

### Optokinetic Responses

A virtual Optokinetic System was used to test visual acuity [20]. The apparatus consisted of four computer monitors around a square testing arena (OptoMotry; Cerebral Mechanics). A sine wave grating was drawn on a virtual cylinder projected in three-dimensional coordinate space on the monitors, and the cylinder was rotated at a constant speed (12°/s). The testing procedure consisted of placing an unrestrained mouse onto a platform in the center of the arena. A video camera provided real-time video feedback from above, and the position of the head on each frame was used to center the hub of the cylinder continually at the mouse’s viewing position. On each trial, an experimenter judged whether the mouse made tracking movements with its head and neck to follow the drifting grating. The spatial frequency threshold, the point at which animals no longer tracked, was obtained by incrementally increasing the spatial frequency of the grating at 100% contrast. Thresholds for each eye were measured separately by reversing the rotation of the cylinder. Mice ranged in age from P32 to P97.

### Immunohistochemistry

Animals were euthanized in a CO_2_ chamber. The enucleated whole eyes were fixed in 4% PFA for 1 hour. To obtain retinal slices, the eyes were sucrose protected, embedded in OCT, frozen at −20°C and cut at 40 microns using a cryostat. Cut slices were transferred onto glass slides. Wholemount retinas and retinal slices were blocked in PBS with 10% normal goat serum (NGS), 10% normal donkey serum (NDS) and 1% TX-100 for 1 hour at room temperature. Subsequent primary and secondary stainings were done in PBS with 1% NGS, 1% NDS and 0.1% TX-100. We used the following primary antibodies: rabbit anti-melanopsin (UF006, Advanced Targeting Systems, http://antibodyregistry.org/AB_1608077) at 1∶1000 and guinea pig anti VGLUT2 (AB2251, Millipore, http://antibodyregistry.org/AB_1587626) at 1∶1000 dilution (overnight at +4°C). Donkey anti-rabbit Alexa488 at 1∶1000 and goat anti-guinea pig Cy3 at 1∶500 were used as secondary antibodies. Images were acquired on a Pascal confocal microscope (Zeiss) and subsequently processed in Photoshop (Adobe). Mice ranged in age from P90 to P289. It should be noted that VGLUTs are localized primarily in the axonal terminals of glutamatergic neurons. It is therefore not unexpected that VGLUT2 immunoreactivity was less than optimum in the somas of mRGCs. However, in wild-type mice, which have two copies of VGLUT2, VGLUT2 immunoreactivity is detectable in the somas of mRGCs in the whole mount retina. However, in our control mice, which have only one copy of VGLUT2 allele, the VGLUT2 immunoreactivity is very poor. Therefore, we failed to quantify the loss of VGLUT2 in somas of mRGCs in cKO mice as compared to control mice. We further attempted to quantify the loss of VGLUT2 in mRGCs by analyzing VGLUT2 immunoreactivity in their axonal terminals in the SCN (suprachiasmatic nucleus), the major central target of mRGCs. Even though there was a statistically significant decrease of overall VGLUT2 signal in cKO mice (p = 0.006) the effect size was small (11% decrease) probably due to other, non retinal, glutamatergic VGLUT2-expressing projections to the SCN. Given that the VGLUT2 loss in cKO mice was sufficient to essentially abolish mRGC-dependent neonatal responses to light, we think the degree of VGLUT2 loss was significant. However, we cannot quantify the extent of its loss in our mice.

### Multielectrode Array Extracellular Recording from Retina

Multielectrode array (MEA-60, MCS) recordings of light-evoked ganglion cell spiking were acquired and analyzed as described previously [21], [22]. Briefly, the MEA chambers consisted of an array of 60 planar electrodes, each 10 µm in diameter, in eight rows and spaced 100 µm apart for a total array size of 700 µm^2^. Acquired voltage signals were bandpass filtered at 0.1 Hz to 3 kHz and sampled at 50 kHz (MC_Rack, version 2.0; MultiChannel Systems). Offline, action potential waveforms from high-pass filtered data (100 Hz lower cutoff) were detected by threshold crossing. To isolate individual RGCs, action potential waveforms were then clustered based on the first two principal components, as described previously [22]. Cluster contours in principal components space were either manually selected or derived from a k-means algorithm (OfflineSorter, version 3.1; Plexon). The algorithm eliminated outlier waveforms at a threshold of 1.3 times the mean distance from the calculated cluster center. Light stimuli were presented from a monitor (Dell Ultrascan P780; 100 Hz vertical refresh) imaged onto the retinal surface at an approximate intensity of 0.35 W/cm^2^. Mice ranged in age from P7 to P9.

### Statistical Analyses

Tests of statistical significance were determined by Student’s *t*-test or Wilcoxon signed rank test (GraphPad Prism), with the criteria of significance set at *P*<0.05.

## Results

### Selective Removal of Glutamate from Synaptic Vesicles in the Melanopsin-expressing Cells

**Figure 1:**
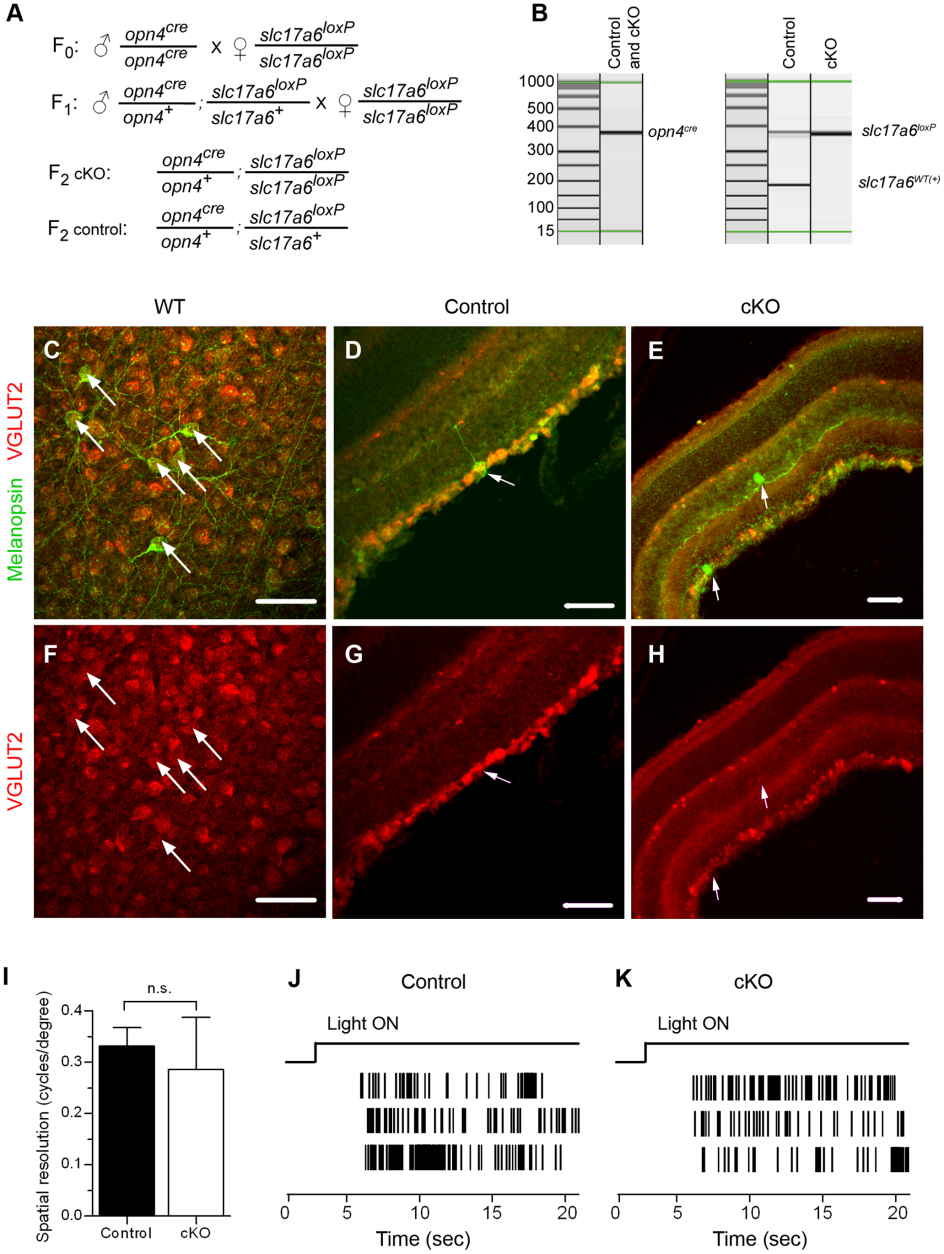
Conditional knockout of VGLUT2 from mRGCs. (**A**) Breeding scheme for obtaining conditional VGLUT2 knockouts (cKO) and controls. (**B**) Mice were genotyped by PCR with allele-specific primers. (**C–H**) VGLUT2 immunoreactivity is detected in melanopsin-immunoreactive retinal ganglion cells in wild-type (WT) (C, F) and control mice (D, G) but not cKO mice (E, H). (C, F) flatmount retina; (D, E, G, H) retinal slices. Arrows point to retinal ganglion cells that are immunopositive for melanopsin. Scale bar: 50 um. (**I**) Mean optokinetic response, a measure of image-forming vision, was not different between control (n = 8 eyes) and cKO (n = 7 eyes) mice (*P* = 0.4, Student’s *t*-test). (**J, K**) Typical action potential responses of neonatal (P8) mRGCs to light in control (J) and cKO (J) retinas. These electrophysiological findings demonstrate that mRGCs in VGLUT2 cKO pups retained their light responses.

To selectively prevent glutamate sequestration into the synaptic vesicles in the axons of melanopsin-expressing retinal ganglion cells (mRGCs), we crossed mice with conditional VGLUT2 allele (*slc17a6^loxP^*/*slc17a6^loxP^* [12]) to mice with cre recombinase in place of the melanopsin gene (*opn4^cre^*/*opn4^cre^* [17]). Previous studies using this same recombination approach demonstrated electrophysiologically its efficacy in eliminating synaptic transmission from VGLUT2-expressing neurons [11], [12]. Figure 1A describes the breeding scheme we utilized for these present studies. For all experiments, we compared control mice that possess one copy of *opn4^cre^* gene and one copy of the floxed *slc17a6* gene (*opn4^cre^*/+; *slc17a6^loxP^*/+) with littermate conditional knockouts (cKO) that possess one copy of *opn4^cre^* gene and two copies of the floxed *slc17a6* gene (*opn4^cre^*/+; *slc17a6^loxP^*/*slc17a6^loxP^*). Mice were genotyped by PCR with allele-specific primers [12], [17] (Figure 1B). To validate the removal of VGLUT2 from mRGCs, we analyzed immunostained retinas. VGLUT2 co-localizes with melanopsin in wild type (Figure 1C,F) and control (Figure 1D,G) but not in cKO retinas (Figure 1E,H). Deletion of VGLUT2 in mRGCs did not have a noticeable effect on the level of VGLUT2 expression in other RGCs.

The cKO mice retained many normal visual functions. Loss of glutamatergic signaling from mRGCs did not affect their intrinsic photosensitivity as detected by the multielectrode array recordings done at the same age range when photoaversive responses were examined (P7 to P9; Figure 1J,K). Visual signaling from other retinal ganglion cells was not discernibly affected as optokinetic responses in control (n = 8 eyes in 4 mice) and cKO mice (n = 7 eyes in 4 mice) were not statistically different (*P* = 0.4) (Figure 1I). There was no detectable difference in the average weight between control and cKO mice (*P* = 0.25). Two out of sixteen cKO mice had ataxia with a persistent head tilt. Veterinary inspection revealed no ear infection. The rest of cKO mice and all control mice (n = 15) had no noticeable anatomical or neurological abnormalities. The two cKO mice with ataxia were included in all tests except optokinetic responses.

### Glutamatergic Signaling from mRGCs is Required for Photoaversion and Light-evoked Ultrasonic Vocalizations in Neonatal Mice

**Figure 2:**
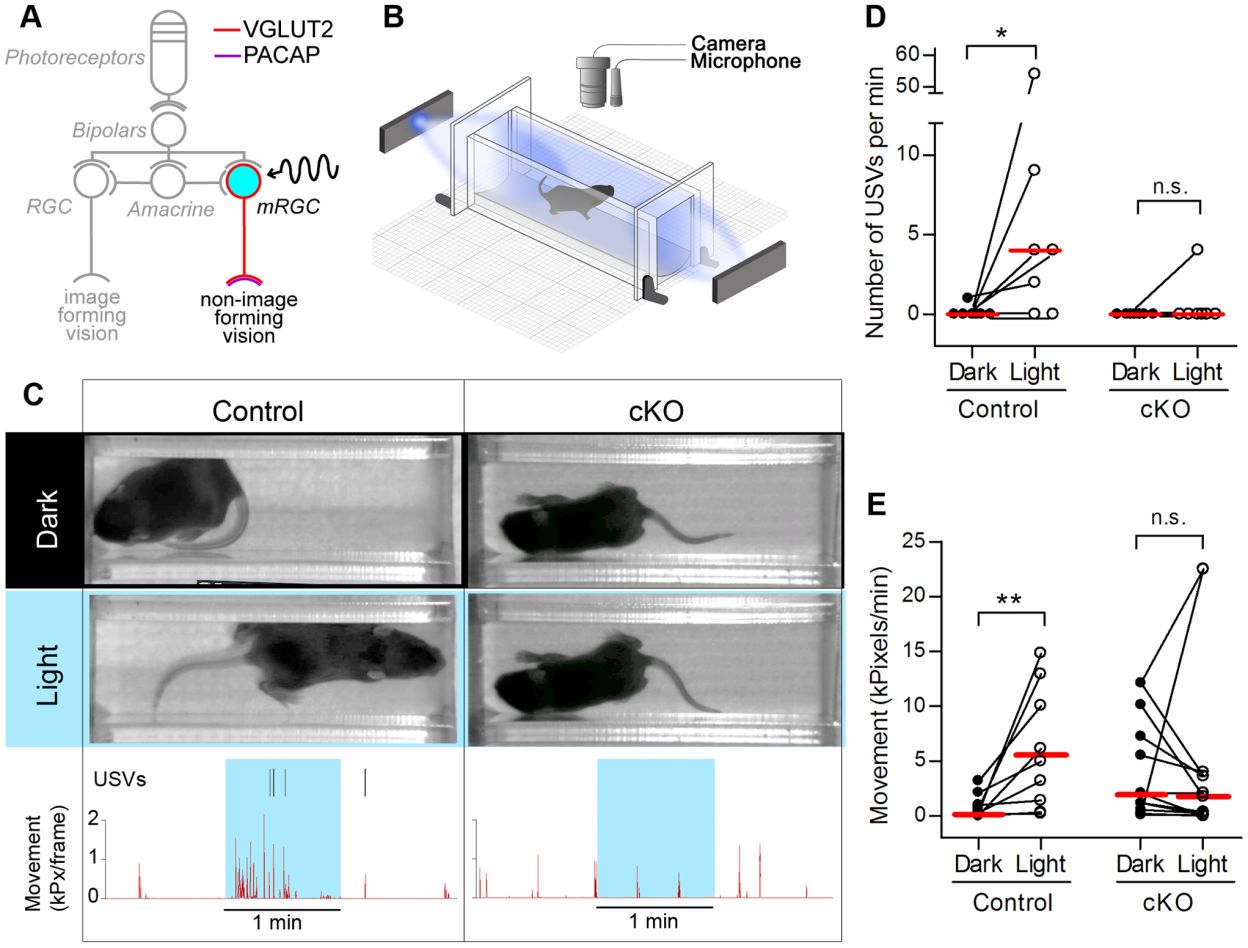
Glutamatergic signaling from mRGCs is required for photoaversion and light-evoked ultrasonic vocalizations in neonatal mice. (**A**) Schematic of photoreception and visual pathways in the retina of neonatal mice. Until postnatal day 10, mice rely on intrinsic photoreception in mRGCs for light-mediated functions as visual signaling from cones, rods and bipolar cells has not yet matured. After P10, visual signals from rods and cones excite RGCs including mRGCs. The schematic shows that neurotransmission from mRGCs relies on glutamate and PACAP. (**B**) Experimental setup to test neonatal photoaversion (see methods). (**C**) Examples of movement and vocal responses to light in control and in cKO mice. Exposure to light evoked increased movement and distress USVs in the control but not in the cKO mouse. The timing of individual USVs is marked by ticks at the top of the graph. Locomotive movement is presented as the amount of net movement in kiloPixels per frame (see Methods). (**D, E**) Summary of light-induced responses. The number of USVs (D) and the amount of net movement (E) significantly increased in the control mice (n = 7 for USVs and n = 9 for movement) but not in the cKO mice (n = 7 for USVs and n = 10 for movement). Red lines are median values. Asterisk (*) indicates *P*<0.05; (**) indicates *P*<0.01; (n.s.) indicates *P*>0.05 (Wilcoxon signed rank test).

We previously showed that until P9, neonatal mice rely on intrinsic photosensitivity of mRGCs to turn away from light (negative phototaxis [1]) and to vocalize in response to light (light-induced ultrasonic vocalizations [2]). However, it is not known if these neonatal responses to light depend on glutamatergic or peptidergic transmission from mRGCs. MRGCs are known to express both VGLUT2 and PACAP [6] (Figure 2A). We therefore asked if removal of glutamatergic transmission affects these responses. Using the previously described testing setup [1], [2] (Figure 2B), we tested movement and vocal responses of P6–P9 control and cKO pups to light. Individual pups were dark adapted and transferred under dim red light into the testing chamber and allowed to acclimate in the dark until isolation-induced calls and locomotor activity significantly diminished. We quantified light-evoked behavioral responses by recording movement and 62-kHz ultrasound vocalizations (USVs) during one min in the dark followed by one min in the light (Figure 2C and Movie S1). During the 1-min exposure to light, control mice showed a significant increase in movement (n = 9) and the number of USVs (n = 7, Figure 2C–E). In contrast, cKO mice did not exhibit an increase in movement (n = 10) or the number of USVs (n = 7, Figure 2C–E, Movie S2). These results demonstrate that VGLUT2 in mRGCs is required for neonatal expression of negative phototaxis and light-induced USVs.

### Removal of Glutamatergic Signaling from mRGCs Unmasks Secondary Signaling Pathway for Pupillary Light Responses

**Figure 3:**
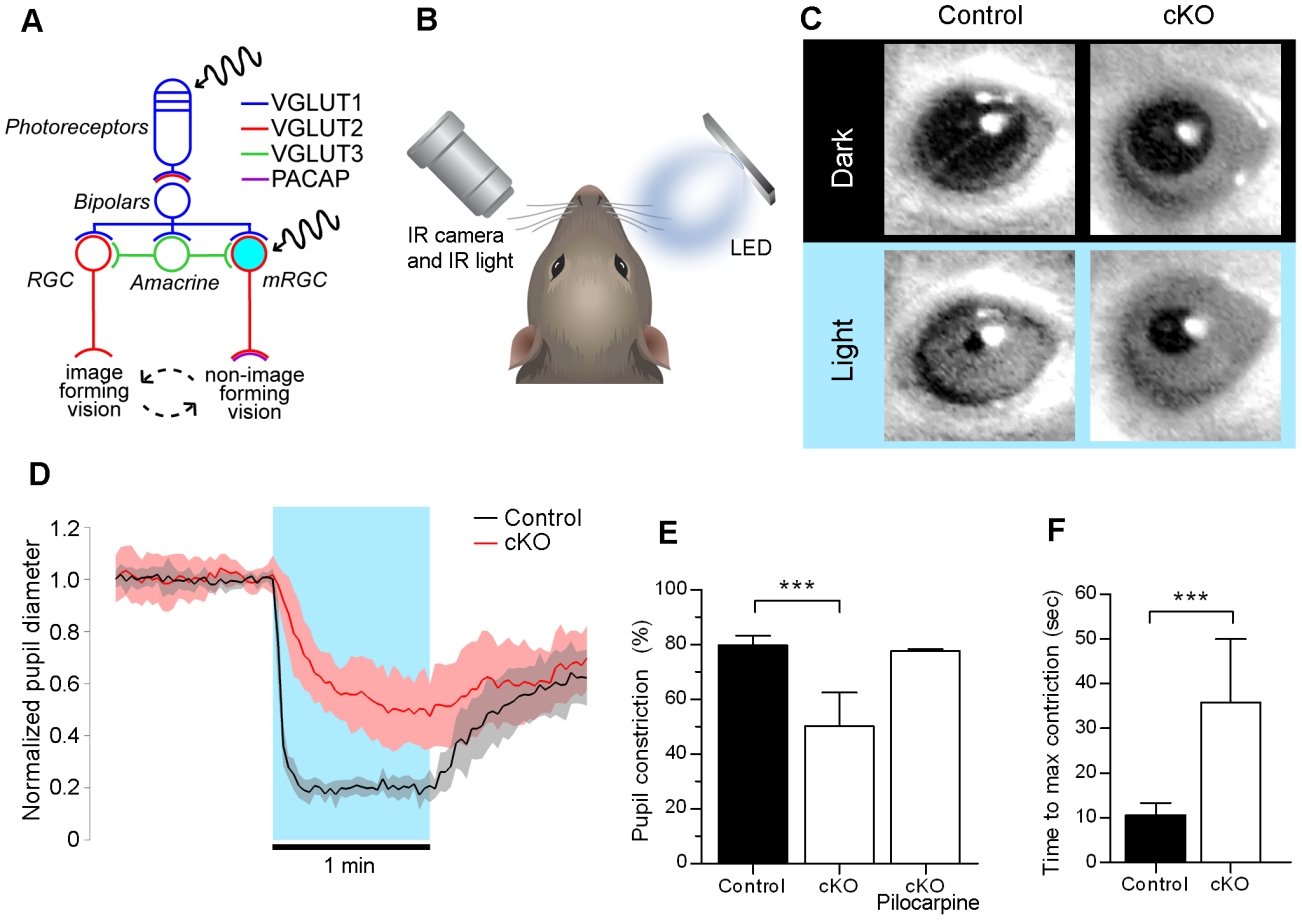
Removal of glutamatergic signaling from mRGCs unmasks secondary signaling pathway for pupillary light responses. (**A**) Schematic of photoreception in the retina of adult mice. Visual signals from photoreceptors are relayed to mRGCs and other retinal ganglion cells via VGLUT1-dependent signaling. MRGCs signal to brain via VGLUT2-dependent signaling and PACAP. (**B**) Experimental setup to test the pupillary light reflex (PLR). Adult mice were dark adapted and then placed into a restraining chamber. The consensual PLR was monitored with an infrared camera. (**C**) Typical PLR responses in control and cKO mice. (**D**) Mean normalized pupil diameter in control and cKO mice during 1 min before light onset, during 1 min light and during 1 min after the light was turned off. Pupil diameter was measured every 2 sec and then normalized to 10-sec baseline before the light onset. Shaded areas mark one standard deviation from the mean. (**E**) Percent maximum constriction in response to 1 min light. Pupils constricted on average 80% in control mice (n = 8) but only 50% in cKO mice (n = 15). Error bars mark one standard deviation from the mean. Asterisks (***) indicate *P*<0.001 (Student’s *t*-test). The incomplete constriction in cKO mice was not due to defects in the iris sphincter muscle or its cholinergic innervation as topical application of pilocarpine constricted the cKO pupils to the control levels (n = 4). (**F**) Mean time to maximum constriction during 1 min after light onset was 11 sec in control mice (n = 8) and 36 sec in cKO mice (n = 15). Error bars mark one standard deviation from the mean. Asterisks (***) indicate *P*<0.001 (Student’s *t*-test).

In adult retina, light is absorbed by mRGCs and by photoreceptors that signal via bipolar cells to both mRGCs and conventional retinal ganglion cells (Figure 3A). To test the role of VGLUT2-dependent glutamatergic transmission from mRGCs on the pupillary light reflex (PLR), we measured light-induced changes in the pupil diameter. Individual adult mice were placed into a restraining chamber in darkness and allowed to acclimate for 4–10 min. A bright blue LED directed at the right eye was then turned on (power flux 35 mW/sq cm) for one min. Consensual pupillary constriction in the infrared-illuminated left eye was recorded with an infrared camera (Figure 3B and Movie S3). Typical responses are illustrated in Figure 3C. A summary of normalized responses is illustrated in Figure 3D. One min light stimulation produced an average 80% constriction in controls (n = 8), but only 50% constriction in cKO mice (n = 15) (Figure 3E and Movie S4). The incomplete pupil constriction in cKO mice was not due to the defect in the iris sphincter muscle or its cholinergic innervation as the application of 1% Pilocarpine, a muscarinic agonist, constricted the pupil to the control levels (Figure 3E). Additionally, PLRs were noticeably slower in cKO than in control mice (40 sec vs. 10 sec) (Figure 3F). This slower and incomplete response in cKO mice is due to some other secondary neurotransmission from mRGCs, which we speculate may be PACAP (see Discussion).

We observed large variability of pupil sizes in cKO mice at baseline in darkness. However, the mean pupil size in the control and cKO mice were not statistically different (p = 0.07). We tried various pharmacological manipulations of sympathetic and parasympathetic innervation of the iris to determine the nature of this variability but failed to determine its origin. These observed variations of pupil size might be due to developmental compensation at the level of innervation of the iris or central mechanisms mediating pupil control.

## Discussion

The role of glutamatergic signaling from melanopsin-expressing retinal ganglion cells (mRGCs) remains largely unexplored. In this study, we have shown that in neonatal mice, loss of VGLUT2-dependent glutamatergic signaling from mRGCs abolishes negative phototaxis and light-induced distress vocalizations. We have also shown that in adult mice, loss of VGLUT2-dependent glutamatergic signaling from mRGCs reveals a slow and an incomplete PLR. We conclude that glutamatergic neurotransmission from mRGCs is required for neonatal photoaversion but is complemented by another non-glutamatergic signaling mechanism for the pupillary light reflex in adult mice.

In neonatal mice, visual signaling from photoreceptors (rods and cones) to bipolar cells and from bipolar cells to retinal ganglion cells (RGC) does not emerge until P10 [1]. Until P10, these young mice rely on the melanopsin photopigment for responses to light. We previously showed that removal of melanopsin abolishes locomotor and vocalization responses to light in neonates [1], [2]. In this present study, we show that conditional removal of VGLUT2 from mRGCs leads to the same loss of responses. These results indicate that VGLUT2-dependent glutamatergic signaling from mRGCs is responsible for both negative phototaxis and light-evoked distress vocalizations. Our results also indicate that other neurotransmitters including PACAP, which is co-expressed in mRGCs before birth [23], [5], are not capable of producing acute behavioral responses to light in neonates.

In adults, loss of VGLUT2 expression in mRGCs reveals a much slower and incomplete PLR, which we suggest is due to PACAP neurotransmission from mRGCs. Previous studies have established that mRGCs are the sole conduits of the light signal for the PLR. Selective destruction of mRGCs leads to complete loss of PLRs [4]. Therefore, the residual response in our conditional VGLUT2 knockout mice is due to some other, VGLUT2-independent signaling from mRGCs. VGLUT1 knockout mice, which lack signaling from photoreceptors and bipolar cells and thus only have mRGC-driven responses, have normal PLRs [3]. Previous studies have failed to identify the expression of other vesicular glutamate transporters in RGCs [8]–[10]. Therefore, this incomplete slow PLR response, unmasked by removal of glutamatergic signaling, is due to complementary non-glutamatergic signaling from mRGCs. We suggest that this complementary signaling is carried out by PACAP. PACAP is present in mRGCs that project to the olivary pretectal nucleus [24] and is capable of signaling on the time scale of seconds [14]. Glutamate appears to be the major signaling molecule for the PLR as the removal of PACAP does not have noticeable effects [15]. However, in the absence of glutamatergic signaling studied here, PACAP is capable of relaying light signal for the PLR, albeit incomplete and at a much slower timescale. To fully elucidate the role of PACAP, PLR responses need to be analyzed in VGLUT2 cKO mice with PACAP null allele.

Based solely on the PLR studies we cannot rule out the possibility that the detected PLR in cKO mice is due to incomplete penetrance of cre-dependent excision of VGLUT2 [25]. However, essentially complete abolishment of neonatal responses to light in the cKO mice, which mimics melanopsin-null phenotype, argues against it. We also failed to detect any fast component of PLR in cKO mice (Figure 3D), which we interpret as a lack of glutamatergic signaling from mRGCs.

Our results indicate that mRGCs do not rely on the single neurotransmitter for initiating all the behavioral and physiological responses to light. Scherrer *et al.* [26] demonstrated that VGLUT2-dependent glutamatergic signaling mediated acute pain and injury-induced heat hypersensitivity while peptidergic signaling might have mediated other nociceptive functions carried by the same afferents. Different brain targets of mRGCs might have variable degree of dependence on glutamatergic or peptidergic signaling. For neonatal photoaversive responses, mRGCs are known to activate central amygdala (CeA) and posterior thalamic region (Po), possibly via direct projections to these brain areas [2], whereas for adult PLR, mRGCs relay the light signal via olivary pretectal nucleus [24]. It is possible that for signaling to CeA and Po, mRGCs rely exclusively on glutamate whereas for signaling to olivary pretectal nucleus, mRGCs use both glutamate and PACAP.

## Supporting Information

Movie S1 **Video and audio recording of negative phototaxis and light-induced 62-kHz USVs in a control mouse pup.** Ultrasonic calls are detected at 62-kHz and shifted to audible frequency ranges by heterodyne circuitry. Video recordings were done with an infrared camera. The first min of recording shows the pup in darkness. The second min is during light stimulation. The third segment shows the next min in darkness.
(MOV)

Movie S2 **Video and audio recording of negative phototaxis and light-induced 62-kHz USVs in a cKO mouse pup.** Ultrasonic calls are detected at 62 kHz and shifted to audible frequency ranges by heterodyne circuitry. Video recordings were done with an infrared camera. The first min of recording shows the pup in darkness. The second min is during light stimulation. The third segment shows the next min in darkness.
(MOV)

Movie S3 **Video recording of pupillary light reflex in a control adult mouse.** The first min of recording shows the mouse in darkness. The second min is during light stimulation. The third segment shows the next min in darkness.
(MOV)

Movie S4 **Video recording of pupillary light reflex in a cKO adult mouse.** The first min of recording shows the mouse in darkness. The second min is during light stimulation. The third segment shows the next min in darkness.
(MOV)
